# QuEChERS-高效液相色谱法同时测定化妆品中的维生素K_1_和K_2_及高效液相色谱-串联质谱确证

**DOI:** 10.3724/SP.J.1123.2025.06011

**Published:** 2025-12-08

**Authors:** Qian SUN, Chunxiao CHEN

**Affiliations:** 厦门市食品药品质量检验研究院，福建 厦门 361012; Xiamen Institute for Food and Drug Control，Xiamen 361012，China

**Keywords:** QuEChERS, 高效液相色谱法, 高效液相色谱-串联质谱法, 化妆品, 维生素K_1_, 维生素K_2_, QuEChERS, high performance liquid chromatography （HPLC）, high performance liquid chromatography-tandem mass spectrometry （HPLC-MS/MS）, cosmetic, vitamin K_1_, vitamin K_2_

## Abstract

建立了不同基质化妆品中同时测定维生素K_1_和K_2_的高效液相色谱（HPLC）分析方法和高效液相色谱-串联质谱（HPLC-MS/MS）确证方法。样品先用饱和氯化钠溶液预分散，经正己烷超声提取并通过QuEChERS前处理方法（150 mg硫酸镁、50 mg PSA、25 mg C_18_）净化，在HPLC方法中，采用CAPCELL PAK C_18_ AQ （250 mm×4.6 mm，5 µm）色谱柱，以甲醇-异丙醇（80∶20，体积比）为流动相，在270 nm波长下进行定量测定，HPLC-MS/MS方法经ACQUITY UPLC BEH C_18_ （50 mm×2.1 mm，1.7 µm）色谱柱分离，含0.05% （体积分数）甲酸和5 mmol/L甲酸铵的甲醇溶液作为流动相洗脱，用电喷雾正离子模式（ESI^+^）、多反应离子监测（MRM）扫描方式对目标物进行确证。在HPLC方法中，维生素K_1_和K_2_在0.1~50 μg/mL范围内线性良好（*r*>0.999），检出限均为0.3 μg/g，定量限均为1.0 μg/g，加标回收率为93.2%~104.5% （RSD<5%）。在HPLC-MS/MS方法中，维生素K_1_和K_2_在0.005～0.5 μg/mL范围内线性良好（*r*>0.999），检出限均为0.02 μg/g，定量限均为0.05 μg/g，加标回收率为89.4%~108.2% （RSD<10%）。本研究建立的方法快速简便，灵敏度高，结果准确，适用于不同基质化妆品中维生素K_1_和K_2_的测定。

## Abstract (en)

Vitamins K_1_ and K_2_ are both essential fat-soluble vitamins for the human body. K_1_ was once used in cosmetics for its efficacy in improving dark circles by promoting periorbital circulation. However， due to its potential to cause severe allergic reactions， it is now banned in cosmetics in China and the European Union. K_2_， particularly the MK-7 form， has a similar structure to K_1_ but features a different side chain. It has gained attention for its skin benefits， including soothing， antioxidant， and anti-aging properties. Although there are no reports of its use in cosmetics in China yet， it has been registered three times as a new cosmetic ingredient， indicating significant market potential. Most existing methods for detecting vitamins K_1_ and K_2_ focus on pharmaceuticals and dietary supplements， with few studies addressing their analysis in cosmetics， especially vitamin K_2_. Given the structural similarity between the two compounds， a reliable method for their simultaneous determination in cosmetic products is needed. However， cosmetic matrices are complex and often interfere with analysis. Conventional sample preparation techniques， such as liquid-liquid extraction and solid-phase extraction， are time-consuming and labor-intensive. The QuEChERS method offers a faster， simpler， and more cost-effective alternative. In this study， we selected six common types of cosmetics—water-based liquids， emulsions， creams， gels， powders， and oils， to develop and validate a simultaneous quantification method for vitamins K_1_ and K_2_. A QuEChERS-based sample preparation method coupled with high performance liquid chromatography （HPLC） was developed for the simultaneous quantification of vitamins K_1_ and K_2_ in cosmetics. To enhance specificity and confirmation capability， a complementary method using high performance liquid chromatography-tandem mass spectrometry （HPLC-MS/MS） was also established. Samples were first pre-dispersed with saturated sodium chloride solution， extracted by *n*-hexane under ultrasonication， and purified using QuEChERS pretreatment technique （containing 150 mg MgSO_4_， 50 mg PSA， and 25 mg C_18_）. For HPLC analysis， separation was performed on a CAPCELL PAK C_18_ AQ column （250 mm×4.6 mm， 5 µm） using methanol-isopropanol （80∶20， volume ratito） as the mobile phase， with detection at 270 nm. For HPLC-MS/MS confirmation， an ACQUITY UPLC BEH C_18_ column （50 mm×2.1 mm， 1.7 µm） was employed， with methanol containing 0.05% （volume fraction） formic acid and 5 mmol/L ammonium formate as the mobile phase. Electrospray ionization in positive mode （ESI^+^） and multiple reaction monitoring （MRM） were used for detection. Both HPLC and HPLC-MS/MS methods demonstrated excellent performance for the determination of vitamins K_1_ and K_2_. In the HPLC method， both analytes showed good linearity over the range of 0.1–50 μg/mL （*r*>0.999）， with limits of detection （LOD） and quantification （LOQ） of 0.3 μg/g and 1.0 μg/g， respectively. The spiked recoveries ranged from 93.2%-104.5% with RSDs below 5%. For the HPLC-MS/MS method， linearity was observed in the range of 0.005–0.5 μg/mL （*r*>0.999）， with LOD and LOQ values of 0.02 μg/g and 0.05 μg/g， respectively. Recoveries in this case fell within 89.4%–108.2%， accompanied by RSDs of less than 10%. The method was successfully applied to analyze 30 cosmetic samples spanning six different matrix types. Neither vitamin K_1_ nor K_2_ was detected in any sample. The proposed methodology is rapid， simple， sensitive， and accurate， making it suitable for routine determination of vitamins K_1_ and K_2_ in diverse cosmetic products. It offers reliable technical support for quality control and regulatory compliance and demonstrates the utility of QuEChERS sample preparation for the analysis of other cosmetic ingredients.

维生素K_1_和K_2_都是人体必需的脂溶性维生素，最早发现的K_1_在促进血液凝结和钙质吸收方面发挥着重要作用，其添加在凝胶中可促进眼周循环，有效改善和防止黑眼圈^［1，2］^，故作为功效成分被逐渐运用到化妆品中，但由于使用含K_1_的化妆品会引起严重的过敏反应而对人体健康造成威胁，出于谨慎的安全考虑，维生素K_1_在我国和欧盟国家属于禁用成分^［3，4］^。维生素K_2_与K_1_化学结构相似，区别主要在于甲萘醌3位侧链的差异，根据异戊二烯侧链的长短不同，可分为14种不同的形式，以MK-*n*（*n*指侧链上异戊二烯单位的个数）表示，其中MK-7生物利用度更高、生物活性更强，是最具代表性和应用价值的维生素K_2_形式，目前研究最多且应用最为广泛（分子结构见图1）^［5］^，其在舒缓、抗氧化、皮肤屏障功能修护、改善皮肤老化等方面有独特优势^［6］^，目前，K_2_在国外已被应用于化妆品且上市销售，在我国虽尚无化妆品使用相关报道，但作为化妆品新原料已备案3次（备案号：国妆原备字20230041、20240045、20250045），市场潜力巨大。

**图1:**
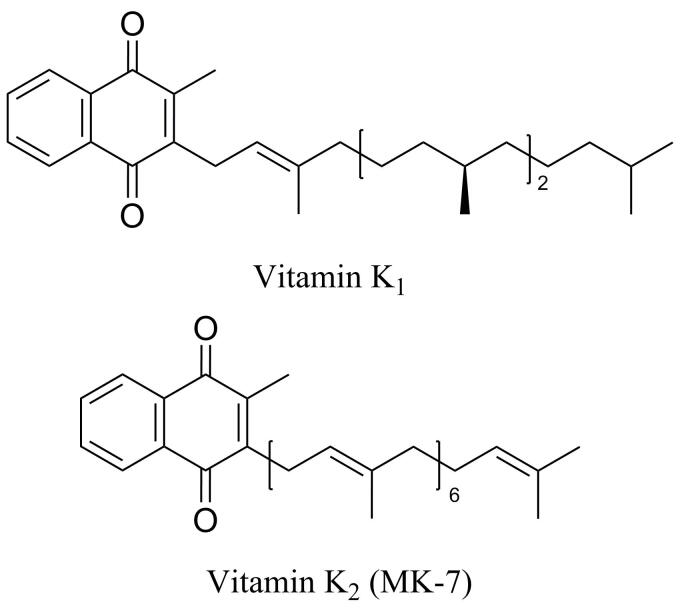
维生素K_1_和K_2_的分子结构

目前，有关维生素K_1_和K_2_的检测研究多集中在药品和保健食品领域，检测方法包括高效液相色谱法（HPLC）^［7-9］^、液相色谱-柱后衍生法^［10-12］^、液相色谱-串联质谱法（LC-MS/MS）^［13-15］^等，但其在化妆品中的测定报道较少，尤其是K_2_，检测方法仍为空白，鉴于K_1_与K_2_结构上的相似性，建立准确可靠、可同时测定化妆品中维生素K_1_与K_2_的高效分析方法十分必要。化妆品基质复杂，常含有大量油脂、乳化剂等干扰物质^［16］^，维生素K_1_和K_2_均为脂溶性维生素，直接提取的前处理方式很可能对实验结果产生干扰，而传统的净化方法液液萃取、硅胶固相萃取柱等^［17-20］^耗时长，检测成本高，步骤烦琐，近年来，QuEChERS法凭借快速、简单、低廉、有效、稳定、安全的优点，已逐渐应用于化妆品检测领域^［21-24］^，该技术可有效提取目标物并吸附基质中的干扰、缩短样品的前处理时间、提高实验效率，非常适用于化妆品中K_1_和K_2_的测定。

基于已有的检测标准和文献报道，本文选用水基、乳液、膏霜、凝胶、散粉、油类为代表基质，旨在建立一套高效、可靠的检测流程。鉴于化妆品分析的实际需求与不同层级实验室的仪器配置差异，本研究采用了“HPLC定量”与“HPLC-MS/MS确证”相结合的分析策略。其中，HPLC方法具有成本低、通量高、易于推广的特点，适用于日常大批量样品的快速筛查与定量分析；而HPLC-MS/MS方法则凭借其极高的选择性和灵敏度，作为最终裁定手段，用于阳性结果的确证，有效规避复杂基质带来的假阳性风险。该策略可兼顾检测效率与结果的确凿性，旨在为化妆品中维生素K_1_和K_2_的测定提供一套完整且实用的技术方案，为国家化妆品相关检验方法的补充提供科学依据。

## 1 实验部分

### 1.1 仪器、试剂与材料

Alliance E2695高效液相色谱仪-2998二极管阵列检测器（美国Waters公司）；Agilent 6410B高效液相色谱-三重四极杆质谱联用仪（美国Agilent公司）；Mettler XS105DE电子天平（瑞士Mettler Toledo公司）；Milli-Q超纯水系统（美国Millipore公司）；XJ-600HE超声波清洗机（方需科技上海有限公司）；MS3 basic涡旋混匀器（德国IKA公司）；3K15冷冻离心机（德国Sigma公司）；1-14高速台式离心机（德国Sigma公司）；EYELA MG-2200氮吹仪（上海爱朗仪器有限公司）；0.22 μm滤膜（天津市津腾实验设备有限公司）。甲醇、乙腈、异丙醇、正己烷（色谱纯，德国默克公司）；氯化钠（分析纯，国药集团化学试剂有限公司）；甲酸、甲酸铵（优级纯，上海阿拉丁生化科技股份有限公司）；水为超纯水；维生素K_1_ （含量99.6%，Dr. Ehrenstorfer公司）；维生素K_2_ （MK-7，含量97.8%，BePure公司）。4种2 mL QuEChERS净化管均购自上海安谱实验科技股份有限公司。

30批试验样品，包括常见的市售水基、乳液、膏霜、凝胶、散粉、油类化妆品，均为厦门市食品药品质量检验研究院的抽检样品。

### 1.2 仪器条件

HPLC条件 色谱柱：CAPCELL PAK C_18_ AQ （250 mm×4.6 mm，5 µm，日本大阪曹達株式会社）；柱温：35 ℃；进样体积：10 μL；流速：1 mL/min；流动相：甲醇-异丙醇（80∶20，体积比）；检测波长：270 nm。

HPLC-MS/MS条件 色谱柱：ACQUITY UPLC BEH C_18_ （50 mm×2.1 mm，1.7 µm，美国Waters公司）；柱温：35 ℃；进样体积：10 μL；流速：0.3 mL/min；流动相：含0.05% （体积分数）甲酸和5 mmol/L甲酸铵的甲醇溶液；离子源：电喷雾（ESI^+^）离子源；检测方式：多反应监测（MRM）模式；干燥气温度：325 ℃；干燥气流速：10 L/min；喷雾压力：241.32 kPa （35 psi ）；毛细管电压：正离子 4 000 V；相应的保留时间及质谱参数详见表1。

**表1:** 维生素K_1_和K_2_的母离子、子离子及其他质谱参数

Vitamin	*t*_R_/min	Precursor ion （*m/z*）	Product ions （*m/z*）	Fragmentor/V	Collision energies/V
K_1_	2.13	451	187^*^， 197	120	20， 22
K_2_	4.10	650	187^*^， 253	180	30， 20

* Quantitative ion.

### 1.3 标准溶液的制备

精密称取维生素K_1_、K_2_对照品各10 mg （精确至0.01 mg），置于100 mL棕色容量瓶中，用甲醇溶解并定容，摇匀，即得100 µg/mL的混合标准储备液，于0~4 ℃避光保存。分别移取不同体积的混合标准储备液用甲醇稀释成质量浓度分别为0.1、0.5、1、5、10、20和50 μg/mL的系列标准溶液供HPLC方法测定用，以及0.005、0.01、0.02、0.05、0.1、0.2和0.5 μg/mL的系列标准溶液供HPLC-MS/MS方法测定用。

### 1.4 样品溶液的制备

称取均匀试样1.0 g （准确至0.000 1 g）于15 mL具塞离心管中，加入2 mL饱和NaCl溶液，涡旋分散1 min，再准确加入10 mL正己烷，涡旋混匀1 min，超声提取20 min （超声功率600 W，超声温度：室温）后取出，8 000 r/min离心10 min，上清液待净化。

吸取1.5 mL上清液于2 mL QuEChERS净化管中（含150 mg硫酸镁、50 mg PSA、25 mg C_18_），涡旋混匀1 min，10 000 r/min离心5 min，准确移取1 mL上清液于40 ℃下氮吹至干，加入1 mL甲醇复溶，经0.22 μm微孔滤膜过滤后进样分析。

## 2 结果与讨论

### 2.1 色谱条件的优化

#### 2.1.1 检测波长的确定

分别取质量浓度为50 µg/mL的维生素K_1_、K_2_标准溶液在200～400 nm波长范围内扫描。结果显示，K_1_、K_2_均在248、270、330 nm处有紫外吸收峰，为保证分析灵敏度且减少基线背景干扰，选择270 nm作为检测波长。结果见图2。

**图2:**
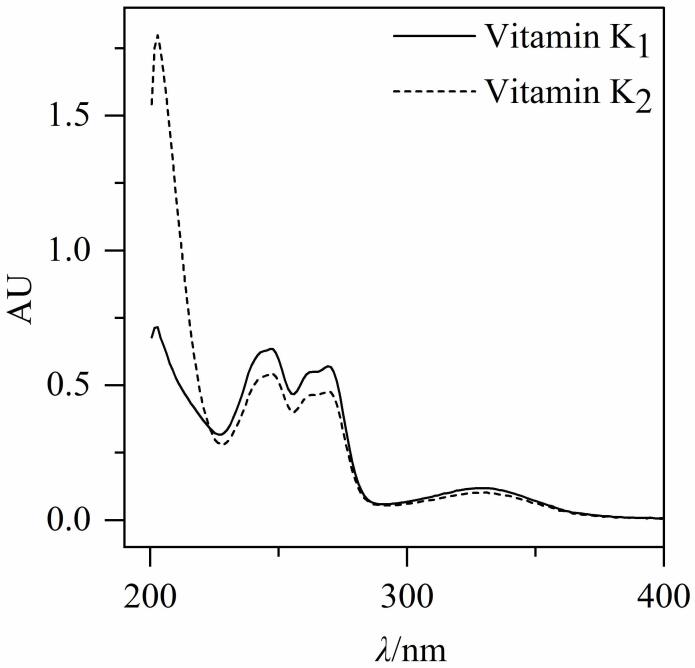
维生素K_1_和K_2_的紫外吸收光谱图

#### 2.1.2 色谱柱的选择

本文对不同品牌、不同型号的5根色谱柱进行了考察，分别为CAPCELL PAK C_18_ AQ、CAPCELL PAK C_18_ MGⅡ、Agilent ZORBAX SB-C_18_、Waters SunFire C_18_、Waters Symmetry C_18_（规格均为250 mm×4.6 mm，5 µm），结果表明，维生素K_1_与K_2_在所有色谱柱上均可获得较好的分离效果，但在CAPCELL PAK C_18_ AQ柱上峰形更窄，保留时间更短，因此采用该色谱柱进行分析。

#### 2.1.3 流动相的选择

维生素K_1_与K_2_属于脂溶性维生素，极性较弱，本文比较了甲醇、乙腈、甲醇-四氢呋喃、甲醇-异丙醇、乙腈-异丙醇5种不同流动相体系的分离效果，结果显示，目标组分在甲醇-异丙醇（80∶20，体积比）流动相条件下峰形更佳，且分析时间更短，因此选用甲醇-异丙醇（80∶20，体积比）为流动相。维生素K_1_与K_2_经色谱条件优化后的HPLC标准溶液图谱见图3。

**图3:**
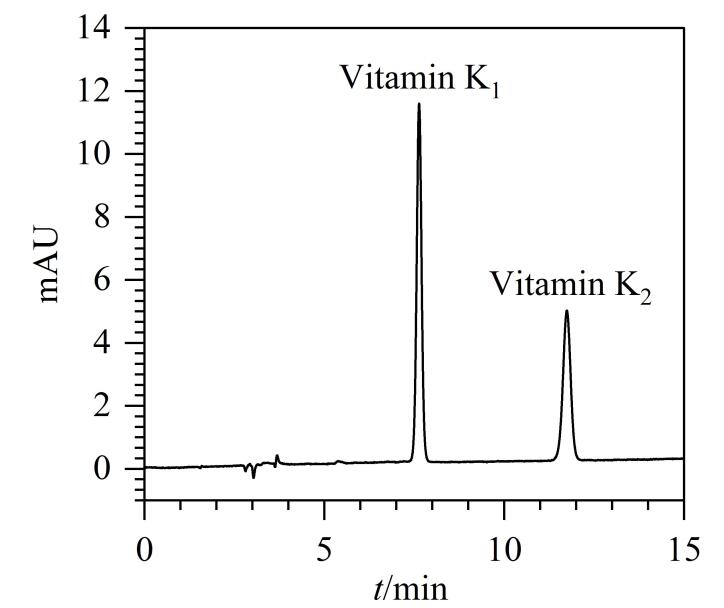
维生素K_1_和K_2_ （5 μg/mL）的HPLC色谱图

#### 2.1.4 质谱确证条件

HPLC方法采用保留时间和光谱定性，很容易受到杂质峰干扰，而HPLC-MS/MS方法通过保留时间和离子对定性，结果更加可靠，如果在样品测试中出现HPLC方法不能确定的干扰情况，可以采用HPLC-MS/MS方法进行确证^［25，26］^。实验结果表明，维生素K_1_、K_2_在ACQUITY UPLC BEH C_18_ （50 mm×2.1 mm，1.7 µm）色谱柱上以及含0.05% （体积分数）甲酸和5 mmol/L甲酸铵的甲醇溶液流动相条件下峰形更佳、灵敏度更高，相较于大气压化学电离（APCI），维生素K_1_、K_2_在ESI^+^模式下响应更强，采用MRM对离子碎片、碰撞能量等质谱参数进行优化，从而确定出最佳质谱条件，如表1所示，质谱确证图谱见图4。在优化后的HPLC-MS/MS实验条件下，通过比较样品溶液与标准溶液色谱峰的保留时间以及母离子和特征子离子信息，则可确证样品中是否存在目标成分。

**图4:**
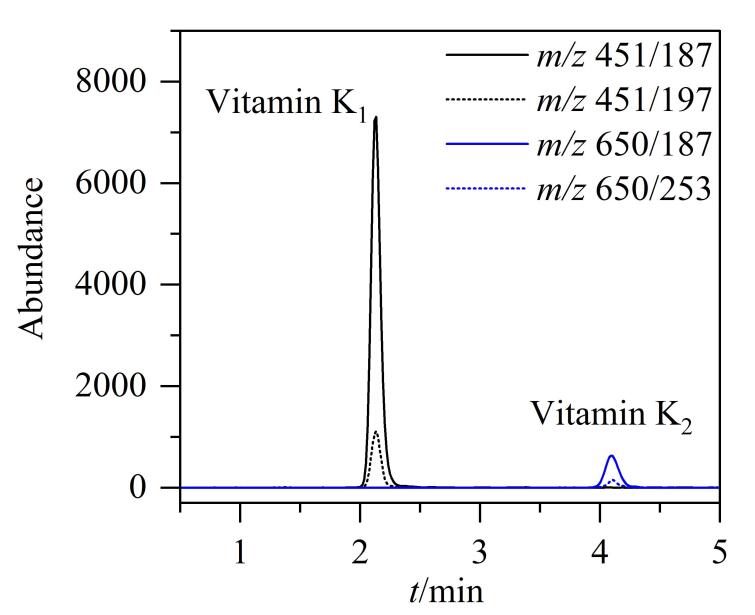
维生素K_1_和K_2_ （0.05 μg/mL）的提取离子色谱图

### 2.2 前处理条件的优化

#### 2.2.1 提取溶剂的选择

维生素K_1_、K_2_的脂溶性较强，常用的提取溶剂包括正己烷、乙酸乙酯、异丙醇等有机溶剂^［27，28］^，由于乙酸乙酯、异丙醇的盐析效果较差，不利于后续使用QuEChERS净化管进行净化操作，故本文选用正己烷作为提取溶剂，对于乳液、膏霜等黏稠型或脂溶性较差的化妆品，采用正己烷直接提取可能很难提取完全，因此考虑先用饱和氯化钠溶液对样品进行分散，再加入正己烷超声提取，经离心分层后，取上清液进一步净化。

#### 2.2.2 净化条件的选择

本文选取了4种不同类型的市售QuEChERS净化管进行试验，分别为：①150 mg硫酸镁、50 mg PSA、50 mg GCB；②150 mg硫酸镁、50 mg PSA；③150 mg硫酸镁、50 mg PSA、25 mg C_18_；④150 mg硫酸镁、25 mg C_18_。分别取6种基质空白样品，在同一添加水平（5 μg/mL）下使用HPLC方法比较维生素K_1_、K_2_的净化后回收率（见表2）。结果显示，使用GCB填料（净化管①）后，维生素K_1_、K_2_的回收率均较低，由于GCB主要对具有平面结构的物质有一定的吸附作用^［29］^，因此该结果可能与K_1_、K_2_具有苯环平面结构有关。净化管③和④都含有C_18_填料，其回收率相近，均高于仅含PSA填料的净化管②，PSA可有效去除有机酸、脂肪酸和极性色素等水溶性杂质，C_18_则对脂肪和脂类等弱极性或非极性杂质具有很强的吸附性能^［30］^。由于本研究的样品经正己烷提取后，样液中除目标成分外，还包括大量弱极性和非极性物质，因此C_18_填料的净化效果优于PSA，也进一步证明，使用QuEChERS技术可以净化除杂、提高目标化合物的提取率。最终，本文选用净化管③（150 mg硫酸镁、50 mg PSA、25 mg C_18_）进行试样净化，净化前后的色谱对比图（以水基类为例）见图5。

**表2:** 采用不同QuEChERS净化管时生素K_1_和K_2_的回收率

Matrix	Vitamin	Recoveries with four tubes/%			
		①	②	③	④
Water-based	K_1_	52.0	95.4	103.7	102.5
	K_2_	29.1	94.3	102.9	101.8
Emulsion	K_1_	66.5	89.5	99.8	98.7
	K_2_	42.7	91.0	104.6	101.5
Cream	K_1_	67.8	94.1	114.0	109.2
	K_2_	51.1	93.9	112.1	108.3
Gel	K_1_	56.3	91.3	104.7	103.9
	K_2_	26.5	90.4	100.2	99.7
Powder	K_1_	57.7	90.9	103.9	100.8
	K_2_	28.6	88.6	101.3	99.9
Oil	K_1_	63.1	95.5	105.7	103.9
	K_2_	55.8	93.8	104.8	105.3

Recovery=post-purification peak area of compound/pre-purification peak area of compound×100%.

**图5:**
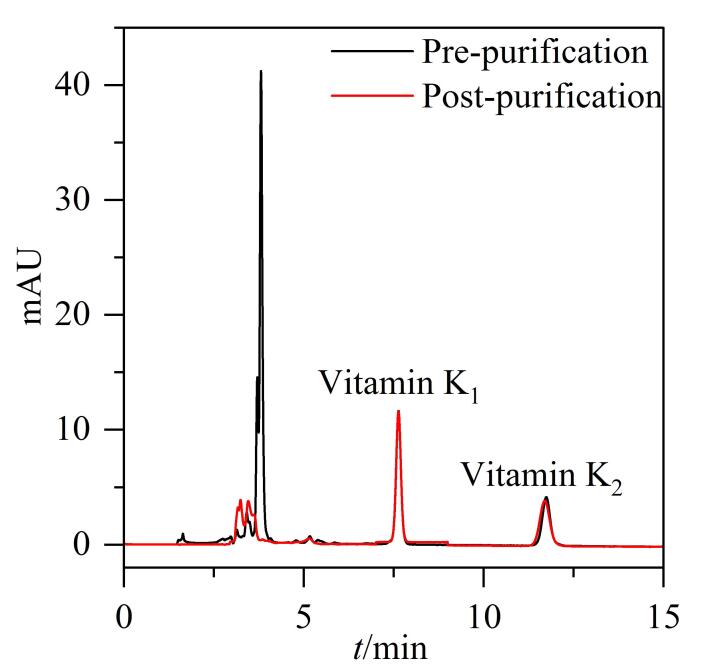
净化前后的色谱对比图（以水基类为例）

### 2.3 方法学验证

#### 2.3.1 线性范围、检出限和定量限

取1.3节中的系列标准溶液，按照1.2节中的测定条件进行测定，以质量浓度（*x，*μg/mL）为横坐标，目标化合物的峰面积（*y*）为纵坐标绘制标准曲线，结果见表3。维生素K_1_、K_2_在0.1~50 μg/mL （HPLC方法）和0.005～0.5 μg/mL （HPLC-MS/MS方法）的范围内线性关系良好，相关系数（*r*）均大于0.999。取空白基质化妆品，加入低浓度标准溶液，按照1.4节进行样品处理，以样品取样量1.0 g、稀释倍数10、3倍信噪比（*S/N*=3）和*S/N*=10分别计算检出限（LOD）和定量限（LOQ），结果见表3。

**表3:** 维生素K_1_和K_2_的线性范围、线性方程、相关系数、检出限、定量限（HPLC法和HPLC-MS/MS法）

Vitamin	HPLC					HPLC-MS/MS				
	Linear range/ （μg/mL）	Linear equation	*r*	LOD/ （μg/g）	LOQ/ （μg/g）	Linear range/ （μg/mL）	Linear equation	*r*	LOD/ （μg/g）	LOQ/ （μg/g）
K_1_	0.1-50	*y*=21803*x*-685.3	1.0	0.3	1.0	0.005-0.5	*y*=1.305×10^6^ *x*+287.5	0.9995	0.02	0.05
K_2_	0.1-50	*y*=14424*x*-1084	1.0	0.3	1.0	0.005-0.5	*y*=2.116×10^5^ *x*+118.2	0.9991	0.02	0.05

*y*： peak area； *x*： mass concentration， μg/mL.

#### 2.3.2 回收率和精密度

称取6种空白基质化妆品各1.0 g，在HPLC方法中，分别加入低（1.5 μg/g）、中（250 μg/g）、高（400 μg/g） 3个水平的标准溶液，按照1.4节制备样品溶液进行回收率试验，同时在HPLC-MS/MS方法中，分别加入低（0.1 μg/g）、中（1.0 μg/g）、高（4.0 μg/g） 3个水平的标准溶液进行回收率试验，每个浓度水平重复6次，计算回收率和相对标准偏差（RSD，*n*=6），结果见表4和表5。结果显示，HPLC法的回收率范围为93.2%~104.5%，RSD为0.3%~4.0%，HPLC-MS/MS法的回收率范围为89.4%~108.2%，RSD为1.2%~7.2%。表明该方法的回收率和精密度结果良好。

**表4:** 维生素K_1_和K_2_在不同基质中的加标回收率及RSD（HPLC方法） （*n*=6）

Vitamin	Spiked/（μg/g）	Recoveries （RSDs） in different matrices/%					
		Water-based	Emulsion	Cream	Gel	Powder	Oil
K_1_	1.5	102.5 （1.2）	100.9 （2.0）	104.5 （3.7）	95.6 （3.4）	100.3 （2.9）	93.9 （3.6）
	250	99.7 （1.3）	97.9 （1.5）	101.1 （1.3）	96.9 （1.0）	98.3 （1.8）	97.5 （1.6）
	400	97.8 （1.0）	101.0 （1.2）	99.6 （1.6）	93.5 （1.1）	101.2 （1.7）	94.6 （0.4）
K_2_	1.5	104.3 （1.6）	102.5 （2.1）	97.7 （2.5）	93.2 （4.0）	98.7 （3.3）	102.3 （2.5）
	250	95.8 （0.7）	96.5 （0.5）	100.1 （1.4）	94.9 （1.4）	97.4 （1.7）	95.2 （1.8）
	400	97.6 （0.9）	100.6 （1.1）	103.4 （1.8）	98.8 （1.9）	100.0 （2.2）	94.4 （0.3）

**表5:** 维生素K_1_和K_2_在不同基质中的加标回收率及RSD （HPLC-MS/MS方法） （*n*=6）

Vitamin	Spiked/（μg/g）	Recoveries （RSDs） in different matrices/%					
		Water-based	Emulsion	Cream	Gel	Powder	Oil
K_1_	0.1	91.6 （5.9）	95.5 （3.1）	91.7 （6.4）	95.8 （4.7）	90.6 （7.2）	96.2 （2.2）
	1.0	97.3 （3.1）	96.7 （3.8）	101.5 （2.6）	97.2 （4.4）	95.3 （3.6）	96.3 （2.5）
	4.0	95.4 （2.6）	96.9 （2.4）	95.8 （1.7）	98.6 （3.5）	95.5 （3.4）	98.0 （2.4）
K_2_	0.1	93.1 （5.7）	94.2 （4.7）	92.1 （5.2）	89.4 （5.6）	92.3 （4.1）	105.9 （2.6）
	1.0	98.4 （3.4）	99.3 （1.8）	99.9 （3.9）	95.3 （4.5）	96.7 （2.4）	99.7 （1.8）
	4.0	103.3 （4.0）	98.8 （1.8）	108.2 （3.7）	96.1 （4.2）	95.3 （2.8）	100.2 （1.2）

### 2.4 HPLC与HPLC-MS/MS法的比较与应用

本研究中，HPLC与HPLC-MS/MS两种方法的方法学参数差异（见表3）揭示了两者检测原理的不同及其在功能上的明确分工。HPLC-MS/MS凭借质谱检测器的高灵敏度和高选择性，实现了更低的检出限（0.02 μg/g）与更窄的线性范围（0.005~0.5 μg/mL），这使其能够可靠地检测并定性痕量目标物，极大避免了假阳性结果的风险。相比之下，HPLC紫外检测法虽灵敏度稍逊，但其方法稳健、线性范围宽（0.1~50 μg/mL），完全满足化妆品中维生素K_1_和K_2_的常规筛查与定量需求，且更具经济性与高通量优势。本研究建立了一种功能互补的分级分析策略：HPLC法作为常规筛查的首选工具，负责快速完成大量样品的初步分析；而HPLC-MS/MS法则作为确证手段，用于对筛查中发现的阳性或可疑样品进行最终裁定。该策略有效整合了两种技术的优势，实现了检测效率与结果可靠性的统一。

### 2.5 样品测定

采用所建立的方法对收集的6种基质（水基、乳液、膏霜、凝胶、散粉、油类）共30批化妆品进行测定，结果显示，所有样品均未检出维生素K_1_、K_2_。

## 3 结论

本文建立了QuEChERS净化技术结合高效液相色谱法同时测定不同基质化妆品中维生素K_1_和K_2_的分析定量和质谱确证方法，针对目标成分的理化性质以及化妆品基质复杂的特点，该方法快速、简便、灵敏度高、适用性强，可提高检验效率和结果的准确性。本方法的建立可为化妆品的质量控制提供有力的技术支持和科学依据，同时，所采用的QuEChERS前处理技术也可为化妆品中其他组分的测定提供新思路。
